# Impact of the COVID-19 Pandemic on Breast Cancer Stage at Diagnosis in a Regional Cancer Center in Poland between 2019 and 2021

**DOI:** 10.3390/jpm12091486

**Published:** 2022-09-11

**Authors:** Maciej Trojanowski, Piotr Radomyski, Krzysztof Matuszewski, Maria Litwiniuk, Ewa Wierzchosławska, Witold Kycler

**Affiliations:** 1 Greater Poland Cancer Registry, Greater Poland Cancer Centre, 61-866 Poznań, Poland; 2 Radiology Department, Greater Poland Cancer Centre, 61-866 Poznań, Poland; 3 Medical Physics Department, Greater Poland Cancer Centre, 61-866 Poznań, Poland; 4 Clinical Oncology Department, Greater Poland Cancer Centre, 61-866 Poznań, Poland; 5 Gastrointestinal Surgical Oncology Department, Greater Poland Cancer Centre, 61-866 Poznań, Poland

**Keywords:** COVID-19, breast cancer, breast cancer stage at diagnosis

## Abstract

The COVID-19 pandemic had a negative effect on oncology healthcare services in Poland, with a reduction in the national breast cancer (BC) screening program coverage rates. This article analyzes the impact of the pandemic on BC stage at diagnosis in a regional cancer center in Poland. Records from BC multidisciplinary team (MDT) meetings that took place in the years 2019–2021 were gathered. BC clinical staging was compared. Age-related subgroups were additionally analyzed to reflect possible screening program disruptions. The total number of BC cases fell by 8% in 2020 compared with 2019, with a 14% fall in the screening age group. In 2021, a stage shift was observed, with stage II BC becoming most frequently diagnosed (as opposed to stage I BC in 2019 and 2020). A statistically significant increase in the number of stage III BC cases was observed in 2021.

## 1. Introduction

### 1.1. Breast Cancer in Poland—Background

According to statistics published by the Polish National Cancer Registry for 2019, breast cancer (BC) was the most frequently diagnosed cancer in women in Poland (22.9% of all cancer cases) and was the second-leading cause of cancer mortality (15.1% of all cancer deaths, surpassed only by lung cancer deaths) [1]. Similar trends were observed in the Greater Poland (GP) region, where BC was ranked first in terms of incidence among women (23.8% of all cancer cases diagnosed) and second in terms of cancer deaths (16.5% of all cancer deaths). BC incidence rates are higher in the GP region (111 per 100,000) compared with average national rates (95 per 100,000)—age-standardized rates using the 2013 European Standard Population. A total of 19,960 BC cases were diagnosed in Poland in 2019, with 1,991 cases diagnosed in the GP region (which represents 10% of all BC cases in Poland) [1,2].

The CONCORD-3 study demonstrated that age-standardized 5-year BC net survival for women diagnosed during 2010–14 in Poland (76.5%) and the GP region (77.4%) was worse than in other EU countries [3]. This could be linked to relatively low screening program participation rates. Population-based BC screening was introduced in Poland in 2007 based on European guidelines. The program offers biennial mammograms to women aged 50–69 with yearly screenings for selected high-risk patients. The participation level hovers around 40% and is markedly lower than the recommended 70% [4].

### 1.2. Impact of the COVID-19 Pandemic on Breast Cancer

The COVID-19 pandemic had a significant impact on the diagnosis and treatment of cancer patients worldwide, especially during the first wave of the pandemic in the first two quarters of 2020 [5,6,7]. Based on the Polish National Health Fund data, a 20% drop in the number of multidisciplinary team (MDT) meetings in oncology was reported in 2020 compared with 2019 [8].

Similar trends were observed among BC patients in other countries [9,10]. Several institutions proposed deferring BC diagnostics and treatment in selected groups of patients due to high numbers of COVID-19 cases [11,12,13]. Regardless of the availability of healthcare services, some patients chose not to seek medical care or take part in cancer screening programs during the pandemic due to various medical and non-medical reasons [14,15].

A rise in advanced BC cases following the COVID-19 pandemic was reported in Italy and Turkey [16,17,18]. Due to pandemic-related disruptions, a small long-term cumulative impact on BC mortality is expected in the USA (to be verified in the following years) [19].

In Poland, restrictions introduced during the COVID-19 pandemic had a significant negative effect on the BC screening program, with a decrease in screening coverage rates in 2020 (a drop from 38.5% in 2019 to 34.5% in 2020) [20,21,22]. The number of screening mammograms in Poland was reduced by 27% in 2020 compared with 2019 [23].

### 1.3. Study Outline

This article analyzes the impact of the COVID-19 pandemic on BC stage at diagnosis in a regional cancer center in Poland (Greater Poland Cancer Centre [GPCC], Poznań, Poland) in the years 2019–2021. GPCC offers BC diagnostics (breast cancer unit, screening program) and treatment (surgery, radiotherapy, clinical oncology), as it is a major site for surgical treatment of BC (54.1% of all BC surgeries in the GP region in the years 2019–2021) [24]. Data from BC MDT meetings were gathered and compared. Patients were divided into several age groups to reflect the potential impact of national BC screening program disruptions.

## 2. Materials and Methods

This is a single-center, retrospective study. Medical records from BC MDT meetings that took place in the years 2019–2021 were collected using the hospital information system (Eskulap, Nexus Polska Sp. z o.o.). The authors had no access to other information on staging stored in patients’ medical history. The Bioethics Committee reviewed the research protocol and waived the need for informed consent, as our study was not classified as a medical experiment.

All cases (3085) of invasive and in situ BC cases were reviewed. Records lacking clinical staging (144) were excluded from the study. Out of a total of 1064 records from 2019, 99 were excluded (9% of cases from 2019). Out of 913 records from 2020, 23 were excluded (2%), and 22 records out of 1108 from 2021 (2%) were excluded—see Table 1.

The study population was divided into four age groups: 0–39, 40–49, 50–69 (BC screening group in Poland), and 70+.

The patients’ age at diagnosis was determined based on the Polish national identification number (PESEL) which includes the date of birth. This was correlated with the date of MDT meetings. Case staging was performed based on the cTNM rules from TNM Classification of Malignant Tumours, 8th edition [25].

Statistical analysis was performed using PQStat v.1.8.4. All variables were assessed for normality using the Shapiro–Wilk test. One-way analysis of variance (ANOVA) GLM followed by Fisher’s least significant difference (LSD) post hoc test, and ANOVA Kruskal–Wallis followed by Dunn–Bonferroni post hoc test were performed to investigate the significance of the difference between the number of breast cancer cases by age group, stage, and year. *p*-values <0.05 were considered statistically significant.

## 3. Results

In 2019, a total number of 965 patients with BC were staged by the MDT. This number dropped by 8% to 890 in 2020 and reached a peak of 1086 patients staged in 2021 (Table 1).

**Figure 1:**
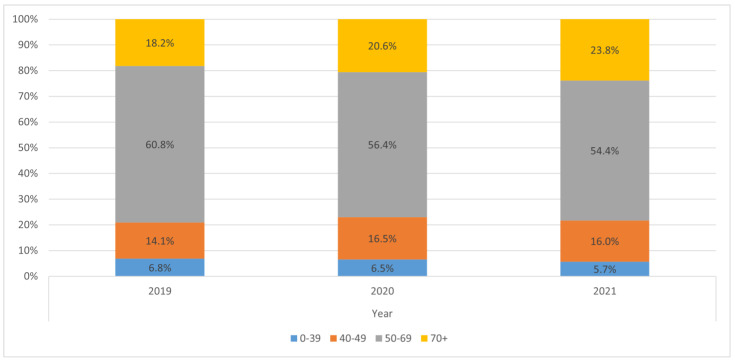
Age group structure of the staged BC cases in years 2019–2021.

The age structure of patients (Table 2, Figure 1) was similar in the analyzed years with a mean age at diagnosis of 60 and a median age of 61–62. The youngest patient was 26 years old and the oldest was 97 (Table 2).

A statistically significant increase in the total number of stage III BC cases was observed in 2021 compared with both 2020 and 2019: 148 cases in 2021 vs. 97 cases in 2020 (*p* = 0.01536) and 90 cases in 2019 (*p* = 0.008829). No statistically significant differences were observed in other BC stages (Table 3).

**Figure 2:**
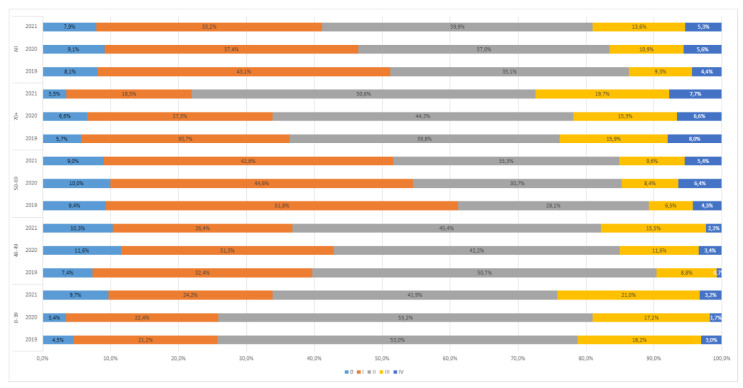
Percentage distribution of BC stages in years 2019–2021 by year and age group.

Table 3 and Figure 2 also show the relative proportion of each BC stage in the years 2019–2021 (expressed as a percentage). In 2019, patients with stage I BC were the most numerous group (43.1% of all patients), followed by stage II BC (35.1%). In 2020, the proportion of stage I BC cases was comparable with stage II BC cases (37.4% and 37.0%, respectively). In 2021, stage II BC patients constituted the majority of cases (39.9%) followed by stage I BC (33.2%).

The percentage of stage I BC cases gradually dropped in the years 2019–2021 (43.1% in 2019, 37.4% in 2020, and 33.2% in 2021). A reverse trend was observed in stage II and III BC cases (stage II BC: 35.1% in 2019, 37.0% in 2020, 39.9% in 2021; stage III BC 9.3% in 2019, 10.9% in 2020, 13.6% in 2021). Stage 0 BC and stage IV BC percentages peaked in 2020; however, these two subgroups were the smallest in the study population (81 cases of stage 0 BC and 50 cases of stage IV BC in 2020).

Table 4 presents more detailed demographic data of patients with their BC stage as determined by the MDT in the years 2019–2021. A statistically significant increase in the number of BC patients, regardless of stage, was observed in the age group 40–49 in 2021 compared with 2019 (174 vs. 136, *p* = 0.02729). Patients who were 50–69 years old were the most numerous group in each year. In this age group, a reduction in the number of cases was observed in 2020 compared with 2019, with an increase in the number of cases in 2021. The differences were not statistically significant (587 in 2019 vs. 502 in 2020, *p* = 0.236495; 587 in 2019 vs. 591 in 2021, *p* = 0.953455; 502 in 2020 vs. 591 in 2021, *p* = 0.217065). It is worth noting that the BC screening age group 50–69 strongly contributed to the drop in the total number of patients analyzed by the MDT in 2020 (a 14% fall in the number of patients aged 50–69 vs. an 8% fall for all age groups combined). There could be a possible link to BC screening disruptions.

An increase in the number of BC cases in the oldest age group (70+) in 2021 may also be connected to reduced screening coverage rates during the pandemic. The differences were not statistically significant (176 in 2019 vs. 259 in 2021, *p* = 0.107431; 183 in 2020 vs. 259 in 2021, *p* = 0.135515)—see Table 5.

No significant differences in patient numbers were seen in the youngest age group, 0–39.

In all four age groups (0–39, 40–49, 50–59, 70+) the greatest number of stage III BC cases was observed in 2021, reflecting the statistically significant increase in the number of stage III BC patients in 2021 in the study population, regardless of age. A drop in the number of stage I BC cases was observed in patients aged 50–69 following the pandemic (304 cases in 2019 vs. 224 cases in 2020 and 252 cases in 2021). The number of stage II, III, and IV BC cases peaked in 2021 in patients over the age of 70 (the number of stage II BC cases in the oldest age group increased to 131 in 2021 vs. 70 cases in 2019).

## 4. Discussion

Our study demonstrates an expected reduction (by 8%) in the number of BC patients discussed at MDT meetings in 2020, which correlates with a reduction in oncological MDT meetings in Poland following the pandemic, reported by Maluchnik et al. [8]. A disproportionately large number of cases from 2019 were excluded from our study due to missing information. The true reduction in the total number of BC cases discussed by the MDT in 2020 was likely higher than that which was reported based on the data available.

Despite the negative effect of the pandemic on BC screening in Poland reported by Andrzejczak et al. [20] and Koczkodaj et al. [21,22], our study did not demonstrate a statistically significant reduction in the number of BC patients aged 50–69, reviewed in 2020 by the BC MDT. This could partly be explained by the relatively small patient population of our single-center study. We did observe a decline in the number of cases in 2020 among patients aged 50–69 (with a p-value of 0,236495). This age group also strongly contributed to the reduction in the total number of cases reviewed by the MDT in 2020. In contrast, the number of patients discussed by the BC MDT in age groups 40–49 and 70+ was higher in 2020 compared with 2019.

The observed reduction in the number of stage I BC cases in the screening age group following the pandemic could potentially be attributed to fewer cases of clinically silent, early BC cases detected by the national screening program (screening coverage rates in Poland dropped to 34.5% in 2020) [22].

The statistically significant increase in the total number of stage III BC cases following the pandemic (in 2021) and changes in the relative distribution of BC stages in the years 2019–2021 outlined by this study comply with similar reports from other countries [11,12,13]. The stage shift and increase in the number of more advanced BC cases could have an impact on the need for different radiotherapy techniques (treatment of metastases and higher BC recurrence rate).

Excluding the youngest patients in our study (0–39, the smallest subgroup) all other age groups’ numbers peaked in 2021, with a statistically significant increase in the number of BC patients aged 40–49. This finding may suggest an ongoing strain on oncology healthcare services following the pandemic. Further research based on the Polish cancer registry’s data into the long-term effects of the pandemic is required, including BC patient mortality and BC survival analyses for cases diagnosed from 2020 in comparison with previous years. These data are not yet available. They will be published at the end of 2022.

### Study Limitations

As a single-center study with a relatively small patient population, our findings may not fully represent the overall impact of the COVID-19 pandemic on BC diagnosis and staging in Poland. However, as population-based data from cancer registries are still being collected, our study is currently a valuable source of information that may serve as a basis for further research.

A large proportion of patients from 2019 (9% of cases) were excluded from our study due to missing data. Unfortunately, we were unable to retrieve the missing information. A new MDT form was introduced in the hospital IT system in the year 2019, which likely had a negative impact on data completeness. The following years showed an improvement in the MDT data collection process. This had an impact primarily on age group comparison, undermining the relative reduction in the number of BC cases diagnosed in 2020. BC stage comparison may also have been affected.

## 5. Conclusions

The number of patients diagnosed with BC in 2020 decreased with the greatest reduction in the number of cases observed in the screening age group. In 2021, a rise in advanced breast cancer cases with a stage shift was observed, which likely results from screening program disruptions observed in 2020. In 2021, stage II BC was the most frequently diagnosed (as opposed to stage I BC in 2019 and 2020). A statistically significant increase in the number of stage III BC cases was observed in 2021. Clinicians should take into account a higher risk of recurrence and metastases due to increased numbers of more advanced BC cases diagnosed after the first year of the pandemic.

## Figures and Tables

**Table 1:** Total number of BC cases reviewed by the MDT team in 2019–2021.

Year	Total Number of Cases	Number of Cases with Clinical Staging
2019	1064	965
2020	913	890
2021	1108	1086
**Total:**	**3085**	**2941**

**Table 2:** Characteristics of BC patients reviewed by the MDT team in 2019–2021.

Patient Characteristics	Year			
	2019	2020	2021	All Cases
**% cases with missing stage data**	9.3	2.5	2.0	4.7
% of in situ cases *	8.1	9.1	7.9	8.3
% of male BC *	0.5	0.7	0.4	0.5
Mean age at diagnosis *	59.8	59.8	60.3	60.0
Minimum age at diagnosis *	26	26	29	26
Maximum age at diagnosis *	96	97	94	97

* staged cases only.

**Table 3:** BC cases with clinical staging, and percentage distribution of each BC stage in years 2019–2021.

	2019		2020		2021		TOTAL	
STAGE	n	%	n	%	n	%	N	%
**0**	78	8.1	81	9.1	86	7.9	**245**	**8.3**
**I**	416	43.1	333	37.4	361	33.2	**1110**	**37.7**
**II**	339	35.1	329	37.0	433	39.9	**1101**	**37.4**
**III**	90	9.3	97	10.9	148	13.6	**335**	**11.4**
**IV**	42	4.4	50	5.6	58	5.3	**150**	**5.1**

**Table 4:** Number of cases grouped by age and stage of BC in 2019–2021.

		2019				2020				2021			
Age gr.		0–39	40–49	50–69	70+	0–39	40–49	50–69	70+	0–39	40–49	50–69	70+
	Stage												
0		3	10	55	10	2	17	50	12	6	18	53	9
I		14	44	304	54	13	46	224	50	15	46	252	48
II		35	69	165	70	32	62	154	81	26	79	197	131
III		12	12	38	28	10	17	42	28	13	27	57	51
IV		2	1	25	14	1	5	32	12	2	4	32	20

**Table 5:** BC cases in years 2019–2021 divided into age group.

	YEAR							
AGE GROUP	2019		2020		2021		**TOTAL**	
	n	%	n	%	n	%	**N**	**%**
**0–39**	66	6.8	58	6.5	62	5.7	**186**	**6.3**
**40–49**	136	14.1	147	16.5	174	16.0	**457**	**15.5**
**50–69**	587	60.8	502	56.4	591	54.4	**1680**	**57.1**
**70+**	176	18.2	183	20.6	259	23.8	**618**	**21.0**
**TOTAL**	965	100.0	890	100.0	1086	100.0	**2941**	**100.0**

## Data Availability

Data available on request in an aggregated form.
